# CX-3543 Promotes Cell Apoptosis through Downregulation of CCAT1 in Colon Cancer Cells

**DOI:** 10.1155/2018/9701957

**Published:** 2018-11-04

**Authors:** Yu-xia Yao, Bao-hong Xu, Yan Zhang

**Affiliations:** ^1^Department of Digestive Diseases, Beijing Luhe Hospital, Capital Medical University, Beijing 101100, China; ^2^Central Laboratory, Beijing Luhe Hospital, Capital Medical University, Beijing 101100, China

## Abstract

**Aim:**

Colon cancer-associated transcript-1 (CCAT1), located in the vicinity of transcription factor c-Myc, was first identified in colon cancer. A small-molecule compound CX3543 (Quarfloxin) selectively targeting Myc G-quadruplexes has entered phase II clinical trials for neuroendocrine carcinomas. The aim of the study was to explore the relationship between CX3543, CCAT1, and cell apoptosis in colon cancer cells.

**Methods:**

Semiquantitative PCR was used to detect the relative expression of CCAT1 in colon cancer (CC) tissues and HT29 cell lines. Real-time PCR (RT-PCR) was also used to investigate the expression of CCAT1 and c-Myc after HT29 cells being treated by CX3543 for 24 h. Cell apoptosis assay and cell proliferation assay were conducted in HT29 cells after being treated by CX3543.

**Results:**

The results showed that the expression of CCAT1 was remarkably increased in CC tissues and HT29 cells compared to controls. CX3543 treatment reduced the expression of c-Myc and CCAT1 and promoted cell apoptosis and inhibited cell proliferation. After the expression of CCAT1 was inhibited by sh-CCAT1 transfection, the cell apoptosis rate was higher than that of control group. After the cells were treated by CCAT1 overexpression plasmid transfection and CX3543, the cell apoptosis rate was lower than that of control group.* In vivo *results showed that CX3543 inhibited the xenograft tumor growth of rats through downregulation of CCAT1.

**Conclusion:**

Our study demonstrated that CX3543 could inhibit the progression of colon cancer by downregulating CCAT1 expression and might be a potential drug for the treatment of colon cancer.

## 1. Introduction

Colon cancer (CC) is a common disease affecting over a million people annually worldwide [1]. Its mortality rate is the third highest among all cancers, leading to approximately 0.6 million deaths annually [2]. Despite major advances in systemic therapy for CC, nearly 50% of patients diagnosed with this common malignancy may recur and die of disease within 5 years of diagnosis and treatment with curative intent. Therefore, it becomes urgent to study the pathogenesis and identify effective risk factors and therapeutic targets of colon cancer. Recently, it is found that long noncoding RNAs (LncRNAs) are closely associated with the carcinogenesis and development of tumor. Colon cancer-associated transcript 1 (CCAT1) is a 2628 bp nucleotide-long, noncoding RNA that maps to chromosome 8q24.21, and was first found to be upregulated in colon cancer [3]. CCAT1 is located in the vicinity of c-Myc, a well-known transcription factor [4].

Preliminary experiments showed that CCAT1 was upregulated in tumor cell lines and tissues obtained from colon cancer (CC) patients and could be activated by c-Myc [5]. Moreover, abnormally expressed CCAT1 promoted cell proliferation and migration [6].

The nuclear transcription factor c-Myc is a member of Myc gene family with multiple functions and located on band q24.1 of chromosome 8 [7]. c-Myc not only is an inducer of cell proliferation, but also has the ability to regulate cell apoptosis via a number of signaling pathways[8, 9]. A small-molecule compound CX3543 (Quarfloxin) selectively targeting Myc G-quadruplexes has entered phase II clinical trials for neuroendocrine carcinomas (NCT00780663) [10]. It could disrupt nucleolin/G-quadruplex complexes in the nucleolus to interact with the biosynthesis of ribosomal RNA in cancer cells and caused apoptosis in cancer cells [11]. It is unknown whether CX3543 could affect the expression of CCAT1 and then induce cell apoptosis.

In this study, we aimed to detect the expression of CCAT1 in CC tissues and colon cancer cells after treatment of CX3543 and to explore the relationship of CX3543, CCAT1, and cell apoptosis in colon cancer cells.

## 2. Materials and Methods

### 2.1. Human Tissue Samples and Cell Culture

One-hundred patients undergoing colon cancer radical resection from May 2015 to May 2016 were recruited in Beijing Luhe Hospital, Capital Medical University (Beijing, China). Tissue samples were obtained from patients undergoing resection for colonic adenoma polyps and carcinoma, normal colonic tissue (n = 20), adenomatous polyps (n = 40), low- and high-grade intraepithelial neoplasia, primary tumor tissues, normal mucosa adjacent to primary tumors (n = 20), and colonic carcinoma (n=20). Specimens were obtained immediately after surgical resection and stored at -80°C for further analysis. All patients had negative histories of exposure to either chemotherapy or radiotherapy before surgery, and there was no other cooccurrence of diagnosed cancers. This study was approved by the Ethical Committee of Beijing Luhe Hospital, Capital Medical University, and has obtained the written informed consent from each patient.

Human HT29 colon cancer cells were obtained from the Cell Bank of Chinese Academy of Medical Sciences (Beijing, China) and were maintained in DMEM medium supplemented with 2 mM L-glutamine, 100 U/ml penicillin, 100 mg/ml streptomycin, and 10% heat-inactivated FBS (v/v) (Thermo Fisher, USA) at 37°C in a humidified atmosphere containing 5% CO_2_.

### 2.2. Semiquantitative PCR Analysis and Quantitative Real-Time PCR Analysis

Quantitative real-time PCR (qRT-PCR) assays were carried out as previously described [12]. The relative expressions of c-Myc and CCAT1 were normalized to GAPDH using the comparative CT method as the manufacturer's instructions (ABI, USA). The primers used in PCR assays were as follows:* GAPDH*_F: 5′-GAAGGTGAAGGTCGGAGTC-3′,* GAPDH*_R: 5′-GAAGATGGTGATGGGATTT-3′;* c-Myc*_F: 5′-CCACAGCAAACCTCCTCACA-3′,* c-Myc*_R: 5′-TCCAACTTGACCCTCTTGGC-3′;* CCAT1*_F: 5′-TCACTGACAACATCGACTTTGAAG-3′,* CCAT1*_R: 5-′GGAGAAAACGCTTAGCCATACAG-3′.

All the qRT-PCR experiments were repeated at least three times with statistical analyses for each individual experimental set. Data analyses for the gene expression were performed using the 2^−ΔΔCt^ method. All values in the experiments were expressed as mean ± standard error of mean (SEM).

### 2.3. Cell Apoptosis Determination

Cells after corresponding treatment were subject to FITC-conjugated Annexin V and PI staining, and apoptotic cells were analyzed by FACS as previously described [13].

### 2.4. CCK-8 Assay

Logarithmically growing HT29 cells were collected and counted. The cells at concentration of 5×10^4^ cells/mL were plated in 96-well plate (100*μ*l/well) and cultured in 5% serum medium without CX3543. In the experimental group, CX3543 at different concentrations (2.5, 5, 10, 20, and 40*μ*g/ml) was added to 5% serum medium. Each treatment was in sextuplicate. A total of 10 *μ*l of CCK-8 was added at 24 h after CX3543 treatment, and the absorbance (OD value) of detected wells was measured using a microplate reader at the wavelength of 450 nm [14]. The survival rate was calculated according to the following formula: survival rate = OD of the experiment well/OD of the control well × 100%. All values in the experiments were expressed as mean ± SEM.

### 2.5. Cell Transfection

The downregulation of CCAT1 in cells was performed using small-interfering RNAs (siRNAs). The upregulation of CCAT1 was performed using pcDNA3.1-CCAT1 overexpression plasmid. The si-CCAT1, si-control, pcDNA3.1-CCAT1 plasmid, and control plasmid were designed and synthesized at Thermo Fisher. The antihuman siRNA specifically targeting CCAT1 was used: 5′-CGG CAG GCA TTA GAG ATG AAC AGC A-3′; and the scramble siRNA was used: 5′-CCU ACG CCA CCA AUU UCG U-3′ and was transfected to HT29 cells using Lipofectamine 2000 (Invitrogen, Carlsbad, CA, USA) according to the manufacturer's instructions, G418 screened stable transfected cell lines and expanded them. Indicated cells were harvested after 72 h of transfection and analyzed as indicated; QRT-PCR was used to detect the expression of CCATI. CCK 8 assay was used to detect the proliferation level of HT29 cells.

### 2.6. Xenograft Tumor Establishment

Twenty-four male Sprague-Dawley rats at 8 weeks of age were purchased from Beijing Vital River Laboratory Animal Technology Co., Ltd. (Beijing, China). The experiment was designed in accordance with the National Institutes of Health guidelines for the use of experimental animals.

To establish the colon xenograft tumor, the rats were divided into 2 groups: the control group and CCAT1 overexpression group. Each group was divided into 3 subgroups and the rats in the 3 subgroups were treated by 10 *μ*g/ml CX3543, 20 *μ*g/ml CX3543, and 40 *μ*g/ml CX3543, respectively. HT29 cells with concentration of 1 × 10^6^ were subcutaneously transplanted into each side of the posterior flank of SD rats. The rats in each group were injected with CX3543 of the corresponding concentration every 3 days. The volume of tumor was determined every week until the fourth week. All of the rats were sacrificed at the 28th day and tumor tissues were isolated to determine the tumor volume and the level of CCAT1.

### 2.7. Statistical Analysis

The GraphPad Prism 6.0 was used to perform the statistical analyses. Data are expressed as the mean ± standard error of the mean (SEM) of three independent experiments. A paired Student's t-test was used to evaluate differences between two groups. Comparison of differences among multiple groups was analyzed with a one-way ANOVA.* p*<0.05 was considered statistically significant.

## 3. Results

### 3.1. Expression of CCAT1 Was Upregulated in the Primary CC Tissues

**Figure 1:**
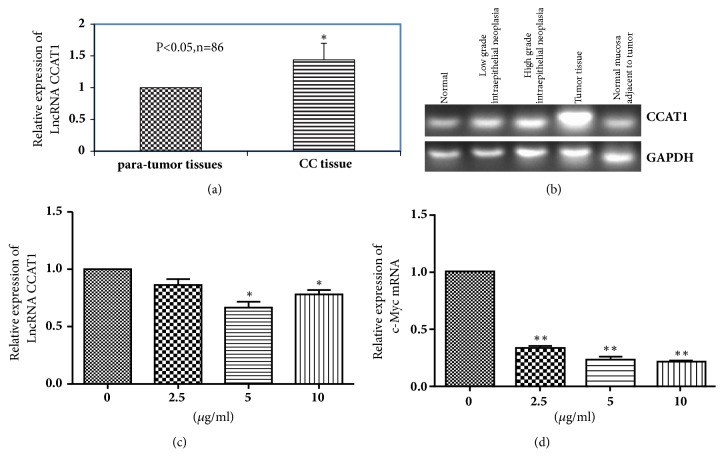
**The expression of CCAT1 and c-Myc was determined using PCR.** (a) The expression levels of CCAT1 in human CC tumor tissues and paratumor tissues relative to GAPDH were determined by RT-PCR. (b) The expression levels of CCAT1 in five kinds of tissues were determined by semiquantitative PCR (n = 86). (c-d) show the expression levels of CCAT1 and c-Myc in HT29 cells, respectively. Data are represented as mean ± SEM. *∗* indicates* p* < 0.05; independent experiment was performed at least three times.

To explore the expression of CCAT1 in the tissues of CC patients, we conducted real-time PCR and semiquantitative PCR assays. The results of real-time PCR indicated that expression levels of CCAT1 in 20 pairs of human CC tissues were significantly upregulated compared to that of the paratumor tissues (p<0.05, Figures 1(a) and 1(b)). The results of semiquantitative PCR showed that the expression of CCAT1 was increased with tumor progression (Figure 1(b)).

### 3.2. CX3543 Suppressed the Expression of c-Myc and CCAT1

CX3543 was selected as a binder of Myc G-quadruplex, and it has been reported that c-Myc could promote CCAT1 transcription by directly binding to its promoter region. Based on the above, HT29 cell line was treated with CX3543 at the concentrations of 2.5 and 5 *μ*g/ml for 24 h. The expression of CCAT1 in the HT29 cells was decreased with the increase of CX3543 concentration compared to the cells without the CX3543 treatment (p=0.0165, Figure 1(c)). Moreover, we showed that the expression of c-Myc also decreased compared to the cells without the CX3543 treatment (p=0.0092, Figure 1(d)).

### 3.3. CX3543 Treatment Inhibited Cell Proliferation

**Figure 2:**
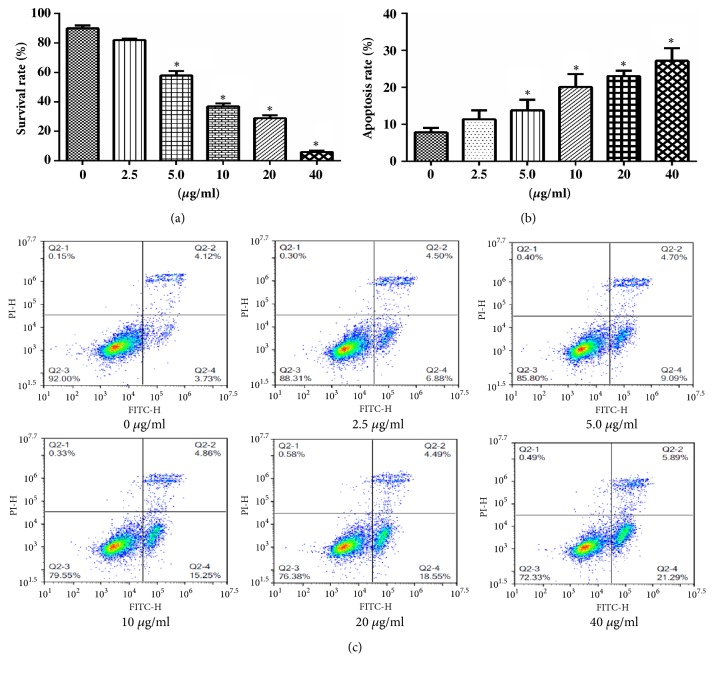
**Effects of CX3543 on proliferation and apoptosis of HT29 cells. **(a) The survival rate of HT29 cells was determined by CCK-8 assay. (b) Cell apoptosis of HT29 cells was statistically analyzed according to the flow cytometer results. (c) Cell apoptosis was detected using flow cytometer after treatment by CX3543 for 24h. Cells were stained with both Annexin V and PI before flow cytometry analysis. Data are represented as mean ± SEM. *∗* indicates* P *< 0.05; independent experiment was performed at least three times.

To evaluate the proliferative effect of CCAT1 in HT29 cells, cell viability was measured using the CCK8 assay following treatment with a number of concentrations of CX3543 (Figure 2(a)). Results show that 2.5, 5, 10, 20, and 40*μ*g/ml of CX3543 could suppress the cell viability of HT29 cells in a dose-dependent manner. The inhibition function of CX3543 was significant for 5, 10, 20, and 40*μ*g/ml of CX3543. The results indicated that CX3543 had an antiproliferative effect against HT29 cells. Therefore, we suggest that CX3543 induced downregulation of CCAT1 and then inhibited cell proliferation.

### 3.4. CX3543 Treatment Promoted Cell Apoptosis

CCAT1 expression in HT29 cells was significantly downregulated after the cells were treated by CX3543 (Figure 1(c)). Based on the expression pattern of CCAT1, the effects of CCAT1 on cell apoptosis were investigated in human CC cell line HT29 using flow cytometry (FACS) with fluorescein isothiocyanate- (FITC-) conjugated Annexin V and propidium iodide (PI) staining. The HT29 cells were treated with CX3543 (2.5, 5, 10, 20, and 40 *μ*g/ml) for 24 h, resulting in inhibited expression of CCAT1. The FACS results indicated that the apoptotic cells (Annexin V-positive population) were dramatically increased in CX3543 treated cells compared to the cells without CX3543 treatment (Figures 2(b) and 2(c)). The results demonstrated that the CX3543 treatment induced cell apoptosis.

### 3.5. Downregulation CCAT1 Promoted Cell Apoptosis

**Figure 3:**
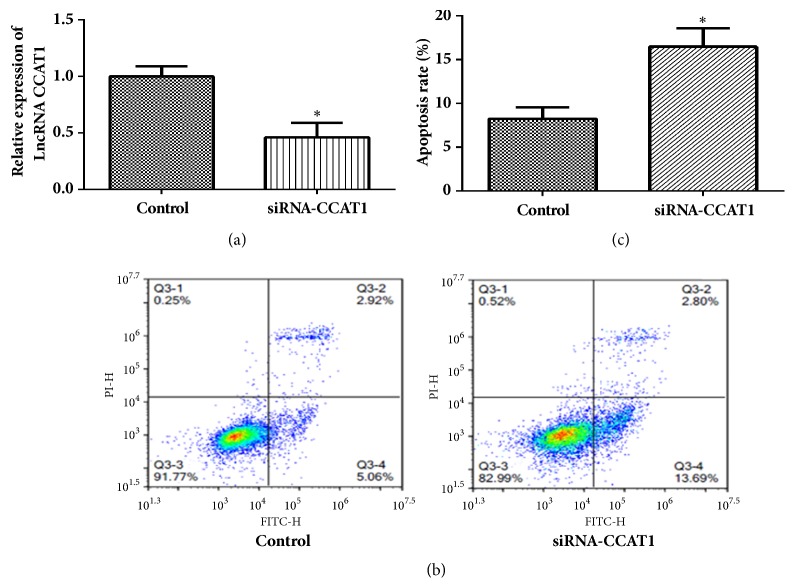
**Downregulation of CCAT1 induced cell apoptosis.** (a) The expression level of CCAT1 was detected using RT-PCR after the HT29 cells were transfected with siRNA-CCAT1. (b) Cell apoptosis was detected using flow cytometer after CCAT1 was downregulated. (c) Cell apoptosis of HT29 cells was statistically analyzed according to the flow cytometer results after CCAT1 was downregulated. Data are represented as mean ± SEM. *∗* indicates* P *< 0.05; independent experiment was performed at least three times.

We have proved that CX3543 could induce cell apoptosis, and we inferred that CX3543 may induce cell apoptosis through decreasing the expression of CCAT1. Therefore, we determined the role of CCAT1 in HT29 cell apoptosis. The HT29 cells were transfected with siRNA-CCAT1 (Figure 3(a)). The FACS results showed that the apoptotic cells in the siRNA-CCAT1 group were more than that of the control group (Figures 3(b) and 3(c)) (8.483 ±1.52 versus 16.32±3.24,* P* = 0.0105). These results indicated that the downregulation of CCAT1 could promote cell apoptosis.

### 3.6. CX3543 Promoted Cell Apoptosis through Downrgulation of CCAT1

**Figure 4:**
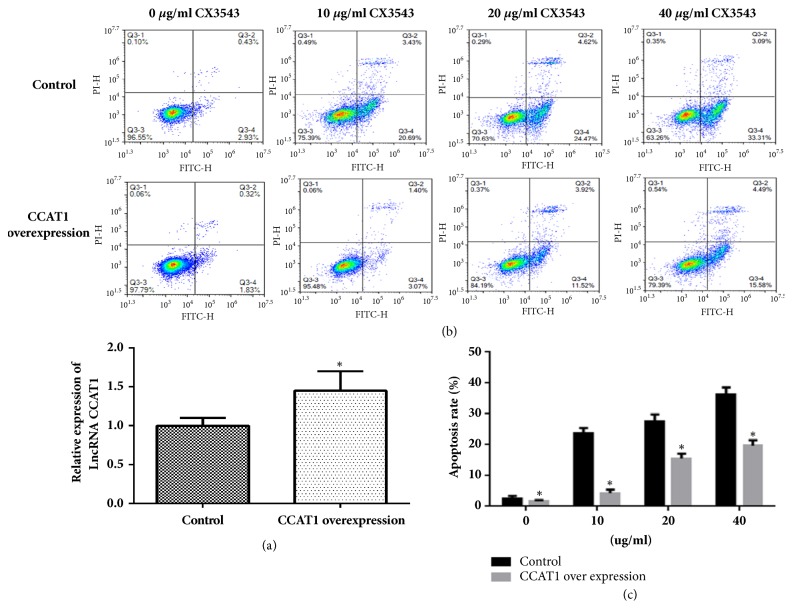
**CX3543 promoted cell apoptosis through downregulation of CCAT1. **(a) The expression level of CCAT1 was detected using RT-PCR after the HT29 cells were transfected with CCAT1 overexpression plasmid. (b) Cell apoptosis was detected using flow cytometer after CCAT1-overexpressed cells were treated with CX3543 at 10, 20, and 40*μ*g/ml, respectively. (c) Cell apoptosis of HT29 cells was statistically analyzed according to the flow cytometer results after CCAT1-overexpressed cells were treated with CX3543 at 10, 20, and 40*μ*g/ml, respectively.

To further explore whether CX3543 promotes cell apoptosis through downregulation of CCAT1, we determined the apoptosis rate of HT29 cells after CX3543 treatment and CCAT1 overexpression. In the experiment, we selected 10, 20, and 40 *μ*g/ml of CX3543 to treat HT29 cells. The expression level of CCAT1 was determined using qRT-PCT (Figure 4(a)). The apoptosis results showed that, at the same concentration of CX3543, the apoptosis rate was significantly higher in cells without CCAT1 overexpression compared to that of the HT29 cells with CCAT1 overexpression (Figures 4(b) and 4(c)). These results proved that CX3543 induced cell apoptosis through CCAT1.

### 3.7. CX3543 Inhibited Xenograft Tumor Growth through CCAT1 In Vivo

**Figure 5:**
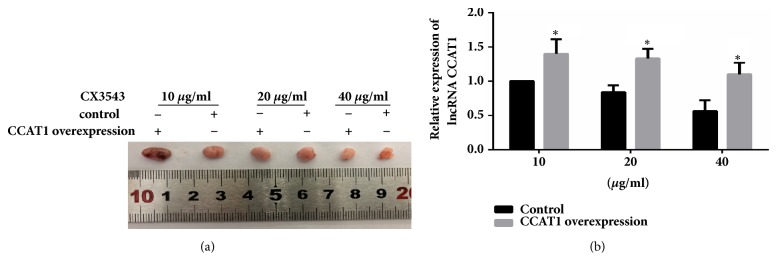
***CX3543 inhibited xenograft tumor growth through CCAT1 in vivo.*** (a) The volumes of xenograft tumors of mice in control group or CCAT1-overexpression group, treated by CX3543 at 10, 20, and 40*μ*g/ml, respectively, were determined. (b) The levels of CCAT1 in xenograft tumors of mice in different groups were determined. Data were represented as mean ± SEM. *∗* indicates* P *< 0.05; independent experiment was performed at least three times.

To further understand the role of CX3543 in the colon tumor, we established xenograft tumor using HT29 cells. The mice in control group were established xenograft tumor using HT29 cells which were stably transfected control plasmid. The mice in CCAT1 overexpression group were established xenograft tumor using HT29 cells which were stably transfected pcDNA3.1-CCAT1 plasmid. Results showed that the tumor volumes were significantly higher in CCAT1 overexpression group than those of the control groups after treatment of 10, 20, and 40 *μ*g/ml of CX3543 (Figure 5(a)). We then detected the level of CCAT1 in tumors. Results showed that the levels of CCAT1 in CCAT1 overexpression group were significantly higher than control group (Figure 5(b), p<0.05). These results showed that CX3543 inhibited xenograft tumor growth through CCAT1* in vivo*.

## 4. Discussion

Because of the high mortality rate of colon cancer, it becomes urgent to discover therapeutic targets and potential drugs for the treatment of colon cancer. In this study we explored the expression of CCAT1 in CC tissues and in CC cells after treatment of CX3543, and whether CX3543 could affect the expression of CCAT1 and then induce cell apoptosis.

Evidence has shown that lncRNAs are involved in human tumorigenesis and misregulated in many cancers and would be valuable biomarkers and therapeutic targets. CCAT1 has been found to be upregulated in many cancers, including gastric carcinoma and colonic adenoma-carcinoma [15–20]. In this study, we first examined the expression of lncRNA CCAT1 in CC tissues and pair-matched noncancerous CC tissues. We found that the expression of CCAT1 was increased in colon cancer compared to paratumor tissues, which was consistent with the previous study [20]. Ye Z et al. have shown that the expression of lncRNA CCAT1 in tumor tissue was significantly higher than that in normal paracarcinoma tissue [20]. We also detected the CCAT1 expression in normal tissue, low-grade intraepithelial neoplasia, high-grade intraepithelial neoplasia, tumor tissue, and normal mucosa adjacent to tumor, and the results showed that the expression of CCAT1 was increased with the progression of cancer. This result is similar to the conclusion drawn by Alaiyan B et al. [4], who explored the expression pattern of CCAT1 in normal colon tissue, adenomatous polyps, primary tumor tissue, normal mucosa adjacent to primary tumor, and lymph node, liver, and peritoneal metastases and concluded that CCAT1 was upregulated across the colon adenoma-carcinoma sequence [4].

CX3543 is a small-molecule compound and may cause apoptosis in cancer cells through disrupting nucleolin/G-quadruplex complexes [10, 11], while whether CX3543 can affect the expression of CCAT1 and then induce cell apoptosis is still unknown. To verify the relationship among CX3543, CCAT1, and colon cancer cell apoptosis, we detected the expression of CCAT1 in colon cancer cell line HT29 after CX3543 treatment. Our results showed that CX3543 significantly downregulated the expression of c-Myc and CCAT1 in HT29 cells. CX3543 also suppressed cell proliferation and accelerated cell apoptosis in HT29 cells. This result is consistent with the conclusion that CX3543 could induce cell apoptosis of cancer cells [11, 21]. Previous studies have shown that CCAT1 could promote cell proliferation and invasion in cells of cancers such as hepatocellular carcinoma, colon cancer, breast cancer, and acute myeloid leukemia [5, 15, 19, 22]. Also we have shown that CCAT1 was downregulated after CX3543 treatment. Based on the above evidences, we conjectured that CX3543 may also accelerate cell apoptosis through downregulating the expression of CCAT1.

To prove the hypothesis, we first detected the role of CCAT1 in colon cancer cell apoptosis after CCAT1 was downregulated using the siRNA. Results indicated that the low expression of CCAT1 promoted the HT29 cell apoptosis, which is consistent with the previous studies [5, 15, 19, 22]. Then we detected the cell apoptosis after CCAT1 was overexpressed and CX3543 treatment, and the results showed that the acceleration of cell apoptosis by CX3543 was abolished by the upregulation of CCAT1, indicating that CX3543 could promote apoptosis by decreasing the expression of CCAT1* in vitro*. We also proved that CX3543 can inhibit tumor growth through downregulation of CCAT1* in vivo*. These results were the highlight of this study because we verified that CX3543 could promote cell apoptosis through downregulated CCAT1 expression. As rRNA biogenesis, CX3543 has been designed to test the safety and tolerability of this drug in patients with advanced solid tumors and lymphomas [23] and this drug has shown low toxicity and now entered phase II clinical trials for neuroendocrine carcinomas [10]. Combining these evidence with our results, CX3543 may be a potential drug for the treatment of colon cancer and CCAT1 may be a therapeutic target of colon cancer.

In the previous studies, CCAT1 was found to be activated by c-Myc in some cancers such as colon cancer, hepatocellular carcinoma, gastric carcinoma, and pancreatic cancer, and c-Myc directly binds to the E-box element in the promoter region of CCAT1, and when ectopically expressed increased promoter activity and expression of CCAT1. Nucleotide substitutions in the E-box element in the promoter region abrogated c-Myc-dependent promoter activation [5, 24–26]. Our results indicated that the expression of c-Myc decreased after HT29 cells were treated with CX3543. However, in this study, we did not determine the role of c-Myc in the mechanism of cell apoptosis promotion by CX3543 with deregulating the expression of CCAT1. We speculated that CX3543 may downregulate the expression of CCAT1 by reducing the expression of c-Myc, which needs to be explored in the next study.

In summary, our findings showed that CCAT1 was abnormally increased in the CC tissues compared with the controls. CX3543 could downregulate the expression of CCAT1 and c-Myc and induce the cell apoptosis through downregulating the CCAT1.

## 5. Conclusion

Our study demonstrated that CX3543 could inhibit the progression of CC by decreasing the expression of CCAT1. CX3543 might be a potential drug for the treatment of colon cancer.

## Data Availability

The data used to support the findings of this study are available from the corresponding author upon request.

## References

[1] Chen W., Zheng R., Baade P. D. (2016). Cancer statistics in China, 2015. *CA: A Cancer Journal for Clinicians*.

[2] Miller K. D., Siegel R. L., Lin C. C. (2016). Cancer treatment and survivorship statistics, 2016. *CA: A Cancer Journal for Clinicians*.

[3] Nissan A., Stojadinovic A., Mitrani-Rosenbaum S. (2012). Colon cancer associated transcript-1: a novel RNA expressed in malignant and pre-malignant human tissues. *International Journal of Cancer*.

[4] Alaiyan B., Ilyayev N., Stojadinovic A. (2013). Differential expression of colon cancer associated transcript1 (CCAT1) along the colonic adenoma-carcinoma sequence. *BMC Cancer*.

[5] He X., Tan X., Wang X. (2014). C-Myc-activated long noncoding RNA CCAT1 promotes colon cancer cell proliferation and invasion. *Tumor Biology*.

[6] Xiang J.-F., Yin Q.-F., Chen T. (2014). Human colorectal cancer-specific CCAT1-L lncRNA regulates long-range chromatin interactions at the MYC locus. *Cell Research*.

[7] Zhang W., Kater A. P., Widhopf G. F. (2010). B-cell activating factor and v-Myc myelocytomatosis viral oncogene homolog (c-Myc) influence progression of chronic lymphocytic leukemia. *Proceedings of the National Acadamy of Sciences of the United States of America*.

[8] Dang C. V. (2012). MYC on the path to cancer. *Cell*.

[9] Hsieh A. L., Walton Z. E., Altman B. J., Stine Z. E., Dang C. V. (2015). MYC and metabolism on the path to cancer. *Seminars in Cell & Developmental Biology*.

[10] Chen B. J., Wu Y. L., Tanaka Y., Zhang W. (2014). Small molecules targeting c-Myc oncogene: promising anti-cancer therapeutics. *International Journal of Biological Sciences*.

[11] Brooks T. A., Hurley L. H. (2010). Targeting MYC Expression through G-Quadruplexes. *Genes & cancer*.

[12] Zhang Y., Zhang Y., Zhang Y.-J., Zhao H.-Y., Zhai Q.-L., Shen Y.-F. (2014). The impact of R213 mutation on p53-mediated p21 activity. *Biochimie*.

[13] Qu G., Liu S., Zhang S. (2013). Graphene oxide induces toll-like receptor 4 (TLR4)-dependent necrosis in macrophages. *ACS Nano*.

[14] Tao M.-Z., Gao X., Zhou T.-J., Guo Q.-X., Zhang Q., Yang C.-W. (2015). Effects of TGF-*β*1 on the Proliferation and Apoptosis of Human Cervical Cancer Hela Cells In Vitro. *Cell Biochemistry and Biophysics*.

[15] Zhu H., Zhou X., Chang H. (2015). CCAT1 promotes hepatocellular carcinoma cell proliferation and invasion. *International Journal of Clinical and Experimental Pathology*.

[16] Xin Y., Li Z., Shen J., Chan M. T. V., Wu W. K. K. (2016). CCAT1: A pivotal oncogenic long non-coding RNA in human cancers. *Cell Proliferation*.

[17] Ma Z. M., Chu F. B., Zhang Y. (2015). Long non-coding RNA CCAT1 promotes gallbladder cancer development via negative modulation of miRNA-218-5p. *Cell Death & Disease*.

[18] Mizrahi I., Mazeh H., Grinbaum R. (2015). Colon cancer associated transcript-1 (CCAT1) expression in adenocarcinoma of the stomach. *Journal of Cancer*.

[19] Zhang X.-F., Liu T., Li Y., Li S. (2015). Overexpression of long non-coding RNA CCAT1 is a novel biomarker of poor prognosis in patients with breast cancer. *International Journal of Clinical and Experimental Pathology*.

[20] Ye Z., Zhou M., Tian B., Wu B., Li J. (2015). Expression of lncRNA-CCAT1, E-cadherin and N-cadherin in colorectal cancer and its clinical significance. *International Journal of Clinical and Experimental Medicine*.

[21] Macaulay R., Rutherford H., Saller J., Scott J., Basanta D., Magliocco A. (2016). Evolutionary advantage of pseudopalisading in diffuse high-grade glioma is unrelated to proliferation or TP53 mutational load. *Neuro-Oncology*.

[22] Chen L., Wang W., Cao L., Li Z., Wang X. (2016). Long Non-Coding RNA CCAT1 Acts as a Competing Endogenous RNA to Regulate Cell Growth and Differentiation in Acute Myeloid Leukemia. *Molecules and Cells*.

[23] Li Q., Xiang J.-F., Zhang H., Tang Y.-L. (2012). Searching drug-like anti-cancer compound(s) based on G-Quadruplex Ligands. *Current Pharmaceutical Design*.

[24] Yang F., Xue X., Bi J. (2013). Long noncoding RNA CCAT1, which could be activated by c-Myc, promotes the progression of gastric carcinoma. *Journal of Cancer Research and Clinical Oncology*.

[25] Yu Q., Zhou X., Xia Q. (2016). Long non-coding RNA CCAT1 that can be activated by c-Myc promotes pancreatic cancer cell proliferation and migration. *American Journal of Translational Research*.

[26] Zhu H.-Q., Zhou X., Chang H. (2015). Aberrant expression of CCAT1 regulated by c-Myc predicts the prognosis of hepatocellular carcinoma. *Asian Pacific Journal of Cancer Prevention*.

